# Supplementary material for: Invasion of the tropical earthworm Pontoscolex corethrurus (Rhinodrilidae, Oligochaeta) in temperate grasslands
Source: PeerJ. 2016 Oct 12;4:e2572. doi: 10.7717/peerj.2572 (PMC5068367; doi:10.7717/peerj.2572)
Supplement: Data S1 [file peerj-04-2572-s001.pdf]

```
R version 3.2.1 (2015-06-18) -- "World-Famous Astronaut"
Copyright (C) 2015 The R Foundation for Statistical Computing
Platform: x86_64-apple-darwin13.4.0 (64-bit)

R is free software and comes with ABSOLUTELY NO WARRANTY.
You are welcome to redistribute it under certain conditions.
Type 'license()' or 'licence()' for distribution details.

  Natural language support but running in an English locale

R is a collaborative project with many contributors.
Type 'contributors()' for more information and
'citation()' on how to cite R or R packages in publications.

Type 'demo()' for some demos, 'help()' for on-line help, or
'help.start()' for an HTML browser interface to help.
Type 'q()' to quit R.

> rm(list=ls())
> library(nlme)
>
> setwd("/Users/paulino/Downloads/Diana")
>
> clima=read.csv(row.names=1,file="clima.csv",header=TRUE)
> suelo=read.csv(row.names=1,file="suelo.csv",header=TRUE)
>
>
> pca.suelo=prcomp(suelo,center=TRUE,scale=TRUE)
>
> plot(pca.suelo)
summary(pca.suelo)
plot(pca.suelo$x[,1:2],pch=19,xlab="PC1 (49%)",ylab="PC2 (21%)")
text(pca.suelo$x[,1:2],rownames(suelo),pos=1,cex=0.50)


pca.clima=prcomp(clima,center=TRUE,scale=TRUE)
plot(pca.clima)
summary(pca.clima)

plot(pca.clima$x[,1:2],pch=19,xlab="PC1 (90%)",ylab="PC2 (9%)")
text(pca.clima$x[,1:2],rownames(clima),pos=1,cex=0.50)


Total=c(17,15,9,14,20,14,6,97,0,
        6, 54, 24, 27, 6, 42, 21,
        30, 5, 12,48)


sites=c(rep("AC",5),rep("N",5),rep("IC",5),rep("LV",5))
sites=as.factor(sites)
```

```
attach(suelo)

fm=lm(Total~sites+ CC + PMP +  pH + MO + N + P + K + cl + sand +
cenizas + CT + N_grass)

summary(fm)

anova(fm)

fm=lm(Total~pca.clima$x[,1]+ CC + PMP +  pH + MO + N + P + K + cl +
sand + cenizas + CT + N_grass)

lTotal=log(Total+1)

fm=lm(lTotal~sites+ CC + PMP +  pH + MO + N + P + K + cl + sand +
cenizas + CT + N_grass)
summary(fm)
anova(fm)

fm=lm(lTotal~pca.clima$x[,1]+ CC + PMP +  pH + MO + N + P + K + cl +
sand + ceni> summary(pca.suelo)
Importance of components:
                          PC1    PC2    PC3     PC4     PC5     PC6
PC7
Standard deviation     2.4544 1.7744 1.4044 0.79114 0.78781 0.50669
0.45402
Proportion of Variance 0.4634 0.2422 0.1517 0.04815 0.04774 0.01975
0.01586
Cumulative Proportion  0.4634 0.7056 0.8573 0.90546 0.95320 0.97295
0.98881
                          PC8    PC9    PC10    PC11     PC12     PC13
Standard deviation     0.2906 0.2072 0.12880 0.03926 0.001359 1.19e-16
Proportion of Variance 0.0065 0.0033 0.00128 0.00012 0.000000 0.00e+00
Cumulative Proportion  0.9953 0.9986 0.99988 1.00000 1.000000 1.00e+00
> plot(pca.suelo$x[,1:2],pch=19,xlab="PC1 (49%)",ylab="PC2 (21%)")
> text(pca.suelo$x[,1:2],rownames(suelo),pos=1,cex=0.50)
>
>
> pca.clima=prcomp(clima,center=TRUE,scale=TRUE)
> plot(pca.clima)
summary(pca.clima)

plot(pca.clima$x[,1:2],pch=19,xlab="PC1 (90%)",ylab="PC2 (9%)")
text(pca.clima$x[,1:2],rownames(clima),pos=1,cex=0.50)


Total=c(17,15,9,14,20,14,6,97,0,
        6, 54, 24, 27, 6, 42, 21,
        30, 5, 12,48)
```

```
sites=c(rep("AC",5),rep("N",5),rep("IC",5),rep("LV",5))
sites=as.factor(sites)

attach(suelo)

fm=lm(Total~sites+ CC + PMP +  pH + MO + N + P + K + cl + sand +
cenizas + CT + N_grass)

summary(fm)

anova(fm)

fm=lm(Total~pca.clima$x[,1]+ CC + PMP +  pH + MO + N + P + K + cl +
sand + cenizas + CT + N_grass)

lTotal=log(Total+1)

fm=lm(lTotal~sites+ CC + PMP +  pH + MO + N + P + K + cl + sand +
cenizas + CT + N_grass)
summary(fm)
anova(fm)

fm=lm(lTotal~pca.clima$x[,1]+ CC + PMP +  pH + MO + N + P + K + cl +
sand + cenizas + CT + N_grass)
anova(fm)

fm_mixed=lme(lTotal~CC + PMP +  pH + MO + N + P + K + cl + sand +
cenizas + CT + N_grass,random=~1|sites)


Pontox=c(0,0,0,0,0,6,3,26,0,1,25,24,27,6,21,0,5,0,0,44)
sites=c(rep("AC",5),rep("N",5> summary(pca.clima)
Importance of components:
                          PC1     PC2     PC3       PC4       PC5
Standard deviation     2.1223 0.68898 0.14565 6.555e-16 2.941e-16
Proportion of Variance 0.9008 0.09494 0.00424 0.000e+00 0.000e+00
Cumulative Proportion  0.9008 0.99576 1.00000 1.000e+00 1.000e+00
>
> plot(pca.clima$x[,1:2],pch=19,xlab="PC1 (90%)",ylab="PC2 (9%)")
> text(pca.clima$x[,1:2],rownames(clima),pos=1,cex=0.50)
>
>
> Total=c(17,15,9,14,20,14,6,97,0,
+         6, 54, 24, 27, 6, 42, 21,
+         30, 5, 12,48)
>
>
> sites=c(rep("AC",5),rep("N",5),rep("IC",5),rep("LV",5))
```

```
> sites=as.factor(sites)
>
> attach(suelo)
>
> fm=lm(Total~sites+ CC + PMP +  pH + MO + N + P + K + cl + sand +
cenizas + CT + N_grass)
>
> summary(fm)

Call:
lm(formula = Total ~ sites + CC + PMP + pH + MO + N + P + K +
    cl + sand + cenizas + CT + N_grass)

Residuals:
       1        2        3        4        5        6        7
8
-0.85364  0.99556  1.68139  0.06196 -1.88526 -0.66507 -3.59383
3.16066
       9       10       11       12       13       14       15
16
 8.82753 -7.72929 -3.94848  3.10968 -3.12069  1.13429  2.82519
2.47798
      17       18       19       20
-3.95127 -2.79614 -1.02579  5.29522

Coefficients:
              Estimate Std. Error t value Pr(>|t|)
(Intercept) 41252.4677 38290.6633   1.077  0.34196
sitesIC        16.6295    14.5312   1.144  0.31628
sitesLV        17.2155    16.1129   1.068  0.34552
sitesN         22.1830    11.5655   1.918  0.12756
CC             -4.0130     1.2355  -3.248  0.03143 *
PMP             9.0778     2.6083   3.480  0.02534 *
pH             16.8988     6.5437   2.582  0.06117 .
MO              2.6670     0.9568   2.787  0.04945 *
N            -317.4383    89.9053  -3.531  0.02421 *
P               0.7413     0.1150   6.447  0.00298 **
K             -26.1554     5.3477  -4.891  0.00810 **
cl             -3.6753     1.3694  -2.684  0.05501 .
sand           -0.9837     0.7046  -1.396  0.23522
cenizas      -420.0784   382.6447  -1.098  0.33393
CT           -708.7483   660.4793  -1.073  0.34366
N_grass        60.4809    15.6673   3.860  0.01814 *
---
Signif. codes:  0 ë***í 0.001 ë**í 0.01 ë*í 0.05 ë.í 0.1 ë í 1

Residual standard error: 8.247 on 4 degrees of freedom
Multiple R-squared:  0.9722,      Adjusted R-squared:  0.8681
F-statistic: 9.338 on 15 and 4 DF,  p-value: 0.02178
```

```
>
> anova(fm)
Analysis of Variance Table

Response: Total
          Df Sum Sq Mean Sq F value  Pr(>F)
sites      3  619.4   206.5  3.0355 0.15571
CC         1  587.3   587.3  8.6357 0.04245 *
PMP        1  124.5   124.5  1.8303 0.24751
pH         1 1275.6  1275.6 18.7548 0.01234 *
MO         1    9.6     9.6  0.1406 0.72672
N          1   21.0    21.0  0.3085 0.60820
P          1 4564.0  4564.0 67.1055 0.00121 **
K          1  518.0   518.0  7.6160 0.05087 .
cl         1  469.2   469.2  6.8993 0.05839 .
sand       1   32.8    32.8  0.4826 0.52547
cenizas    1  164.7   164.7  2.4213 0.19468
CT         1  126.9   126.9  1.8663 0.24366
N_grass    1 1013.5  1013.5 14.9022 0.01814 *
Residuals  4  272.1    68.0
---
Signif. codes:  0 ë***í 0.001 ë**í 0.01 ë*í 0.05 ë.í 0.1 ë í 1
>
> fm=lm(Total~pca.clima$x[,1]+ CC + PMP +  pH + MO + N + P + K + cl +
sand + cenizas + CT + N_grass)
>
> lTotal=log(Total+1)
>
> fm=lm(lTotal~sites+ CC + PMP +  pH + MO + N + P + K + cl + sand +
cenizas + CT + N_grass)
summary(fm)
anova(fm)

fm=lm(lTotal~pca.clima$x[,1]+ CC + PMP +  pH + MO + N + P + K + cl +
sand + cenizas + CT + N_grass)
anova(fm)

fm_mixed=lme(lTotal~CC + PMP +  pH + MO + N + P + K + cl + sand +
cenizas + CT + N_grass,random=~1|sites)


Pontox=c(0,0,0,0,0,6,3,26,0,1,25,24,27,6,21,0,5,0,0,44)
sites=c(rep("AC",5),rep("N",5),rep("IC",5),rep("LV",5))
sites=as.factor(sites)

lm(Pontox~sites)

anova(lm(Pontox~sites))

boxplot(Pontox~sites)
```

```
lPontox=log(Pontox+1)
anova(lm(lPontox~sites))

#Kruskal-Wallis

kruskal.test(Pontox,sites)


> summary(fm)

Call:
lm(formula = lTotal ~ sites + CC + PMP + pH + MO + N + P + K +
    cl + sand + cenizas + CT + N_grass)

Residuals:
        1         2         3         4         5         6         7
8
-0.041068 -0.039744 -0.129601  0.047398  0.163015  0.123129  0.008866
-0.150147
        9        10        11        12        13        14        15
16
-0.234050  0.252201  0.186329  0.257104  0.071166 -0.220014 -0.294586
0.283239
       17        18        19        20
 0.173078 -0.203211 -0.008418 -0.244688

Coefficients:
              Estimate Std. Error t value Pr(>|t|)
(Intercept)  4.402e+01  1.882e+03   0.023  0.98247
sitesIC      1.020e+00  7.144e-01   1.428  0.22660
sitesLV      3.936e-01  7.922e-01   0.497  0.64538
sitesN       7.004e-02  5.686e-01   0.123  0.90790
CC          -2.083e-01  6.074e-02  -3.430  0.02654 *
PMP          4.840e-01  1.282e-01   3.774  0.01953 *
pH           9.200e-01  3.217e-01   2.860  0.04594 *
MO           1.563e-01  4.704e-02   3.324  0.02928 *
N           -1.809e+01  4.420e+00  -4.092  0.01495 *
P            1.844e-02  5.653e-03   3.261  0.03105 *
K           -1.053e+00  2.629e-01  -4.007  0.01604 *
cl          -1.336e-01  6.732e-02  -1.984  0.11820
sand         1.951e-03  3.464e-02   0.056  0.95779
cenizas     -9.710e-01  1.881e+01  -0.052  0.96131
CT          -6.481e-01  3.247e+01  -0.020  0.98503
N_grass      3.559e+00  7.702e-01   4.621  0.00987 **
---
Signif. codes:  0 ë***í 0.001 ë**í 0.01 ë*í 0.05 ë.í 0.1 ë í 1

Residual standard error: 0.4054 on 4 degrees of freedom
Multiple R-squared:  0.9656,      Adjusted R-squared:  0.8368
```

```
F-statistic: 7.495 on 15 and 4 DF,  p-value: 0.03241

> anova(fm)
Analysis of Variance Table

Response: lTotal
          Df Sum Sq Mean Sq F value   Pr(>F)
sites      3 2.7469  0.9156  5.5701 0.065263 .
CC         1 2.0907  2.0907 12.7181 0.023454 *
PMP        1 0.5828  0.5828  3.5451 0.132845
pH         1 1.9053  1.9053 11.5902 0.027166 *
MO         1 0.0020  0.0020  0.0120 0.918032
N          1 0.0012  0.0012  0.0072 0.936305
P          1 5.1139  5.1139 31.1092 0.005065 **
K          1 0.0971  0.0971  0.5909 0.484959
cl         1 1.3530  1.3530  8.2304 0.045520 *
sand       1 0.1181  0.1181  0.7183 0.444444
cenizas    1 0.9375  0.9375  5.7033 0.075318 .
CT         1 0.0222  0.0222  0.1348 0.732076
N_grass    1 3.5100  3.5100 21.3525 0.009875 **
Residuals  4 0.6575  0.1644
---
Signif. codes:  0 ë***í 0.001 ë**í 0.01 ë*í 0.05 ë.í 0.1 ë í 1
>
> fm=lm(lTotal~pca.clima$x[,1]+ CC + PMP +  pH + MO + N + P + K + cl +
sand + cenizas + CT + N_grass)
> anova(fm)
Analysis of Variance Table

Response: lTotal
                 Df Sum Sq Mean Sq F value   Pr(>F)
pca.clima$x[, 1]  1 0.2966  0.2966  1.6429 0.247232
CC                1 4.2343  4.2343 23.4568 0.002871 **
PMP               1 0.5540  0.5540  3.0691 0.130352
pH                1 1.5712  1.5712  8.7038 0.025606 *
MO                1 0.0893  0.0893  0.4946 0.508228
N                 1 0.0111  0.0111  0.0615 0.812476
P                 1 3.7313  3.7313 20.6703 0.003906 **
K                 1 0.1808  0.1808  1.0015 0.355580
cl                1 1.6336  1.6336  9.0495 0.023754 *
sand              1 0.0105  0.0105  0.0584 0.817167
cenizas           1 0.0687  0.0687  0.3805 0.560021
CT                1 0.0667  0.0667  0.3697 0.565458
N_grass           1 5.6070  5.6070 31.0612 0.001415 **
Residuals         6 1.0831  0.1805
---
Signif. codes:  0 ë***í 0.001 ë**í 0.01 ë*í 0.05 ë.í 0.1 ë í 1
>
> fm_mixed=lme(lTotal~CC + PMP +  pH + MO + N + P + K + cl + sand +
cenizas + CT + N_grass,random=~1|sites)
```

```
>
>
> Pontox=c(0,0,0,0,0,6,3,26,0,1,25,24,27,6,21,0,5,0,0,44)
> sites=c(rep("AC",5),rep("N",5),rep("IC",5),rep("LV",5))
> sites=as.factor(sites)
>
> lm(Pontox~sites)

Call:
lm(formula = Pontox ~ sites)

Coefficients:
(Intercept)      sitesIC      sitesLV       sitesN
 -3.178e-15    2.060e+01    9.800e+00    7.200e+00

>
> anova(lm(Pontox~sites))
Analysis of Variance Table

Response: Pontox
          Df Sum Sq Mean Sq F value  Pr(>F)
sites      3 1094.0  364.67  2.6179 0.08665 .
Residuals 16 2228.8  139.30
---
Signif. codes:  0 ë***í 0.001 ë**í 0.01 ë*í 0.05 ë.í 0.1 ë í 1
>
> boxplot(Pontox~sites)
>
> lPontox=log(Pontox+1)
> anova(lm(lPontox~sites))
Analysis of Variance Table

Response: lPontox
          Df Sum Sq Mean Sq F value   Pr(>F)
sites      3 22.523  7.5077   6.288 0.005051 **
Residuals 16 19.104  1.1940
---
Signif. codes:  0 ë***í 0.001 ë**í 0.01 ë*í 0.05 ë.í 0.1 ë í 1
>
> #Kruskal-Wallis
>
> kruskal.test(Pontox,sites)

        Kruskal-Wallis rank sum test

data:  Pontox and sites
Kruskal-Wallis chi-squared = 10.03, df = 3, p-value = 0.01832
```
