# Supplementary material for: Invasion of the tropical earthworm Pontoscolex corethrurus (Rhinodrilidae, Oligochaeta) in temperate grasslands
Source: PeerJ. 2016 Oct 12;4:e2572. doi: 10.7717/peerj.2572 (PMC5068367; doi:10.7717/peerj.2572)
Supplement: Data S2 [file peerj-04-2572-s002.pdf]

First Run:

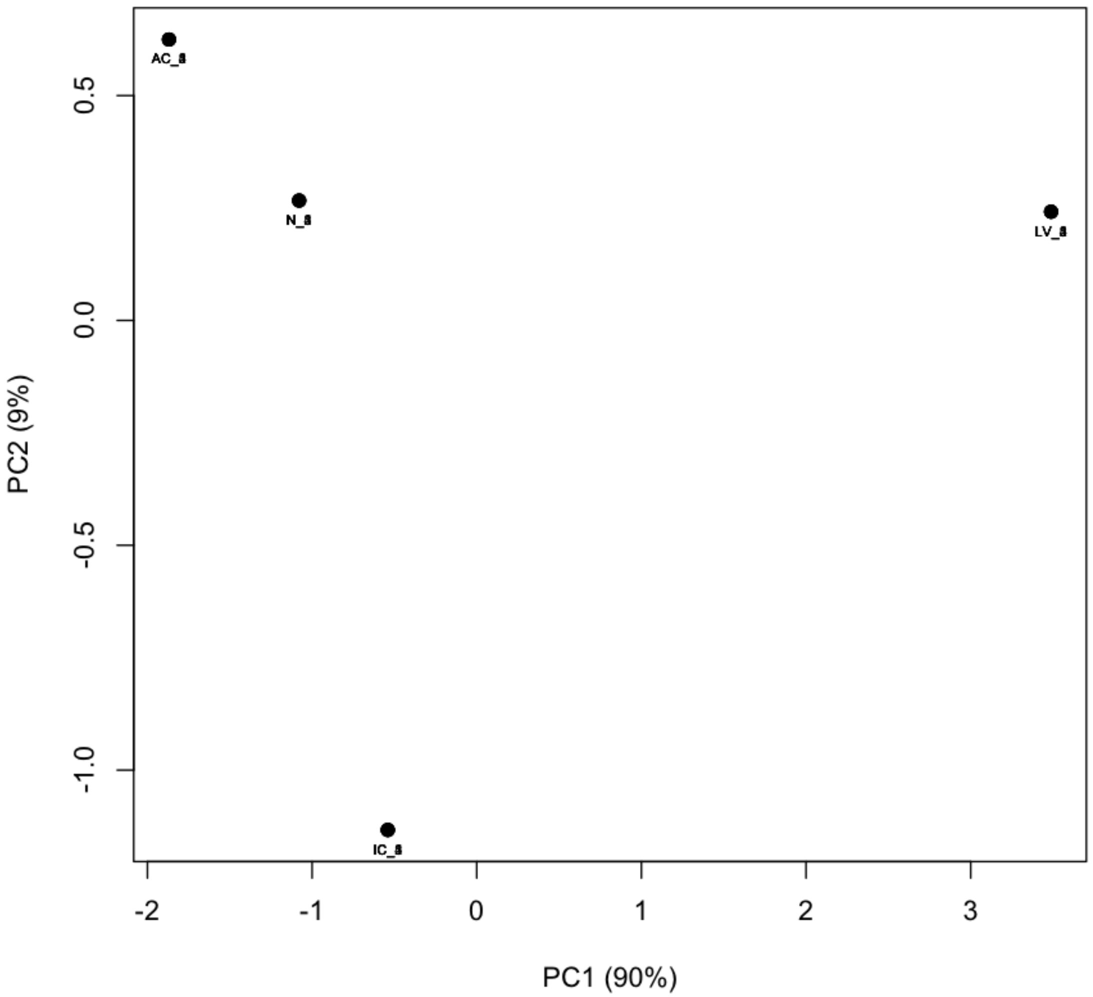

lm(Total~sites+ WCP + PPP + pH + MO + N + P + K + cl + sand + ashes + CT + N_grass)

> anova(fm)
Analysis of Variance Table

Response: Total

	Df	Sum Sq	Mean	Sq F value	Pr(>F)
sites	3	619.3	206.4	3.0355	0.15571
WCP	1	587.3	587.3	8.6357	0.04245 *
PPP	1	124.5	124.5	1.8303	0.24751
pH	1	1275.6	1275.6	18.7548	0.01234 *
MO	1	9.6	9.6	0.1406	0.72672
N	1	21.0	21.0	0.3085	0.60820
P	1	4564.0	4564.0	67.1055	0.00121 **

K	1	518.0	518.0	7.6160	0.05087
cl	1	469.2	469.2	6.8993	0.05839
sand	1	32.8	32.8	0.4826	0.52547
ashes	1	164.7	164.7	2.4213	0.19468
CT	1	126.9	126.9	1.8663	0.24366
N_grass	1	1013.5	1013.5	14.9022	0.01814 *
Residuals	4	272.1	68.0		

Signif. codes: 0 ‘***’ 0.001 ‘**’ 0.01 ‘*’ 0.05 ‘.’ 0.1 ‘ ’ 1

Second Run

fm=lm(Total~sites+ WCP + PPP + pH + MO + N + P + K + cl + sand + ashes + CT + N_grass)

Analysis of Variance Table

Response: Total

	Df	Sum Sq	Mean Sq	F value	Pr(>F)
sites	3	619.3	206.4	3.0355	0.15571
WCP	1	587.3	587.3	8.6357	0.04245 *
PPP	1	124.5	124.5	1.8303	0.24751
pH	1	1275.6	1275.6	18.7548	0.01234 *
MO	1	9.6	9.6	0.1406	0.72672
N	1	21.0	21.0	0.3085	0.60820
P	1	4564.0	4564.0	67.1055	0.00121 **
K	1	518.0	518.0	7.6160	0.05087 .
cl	1	469.2	469.2	6.8993	0.05839 .
sand	1	32.8	32.8	0.4826	0.52547
ashes	1	164.7	164.7	2.4213	0.19468
CT	1	126.9	126.9	1.8663	0.24366
N_grass	1	1013.5	1013.5	14.9022	0.01814 *
Residuals	4	272.1	68.0		

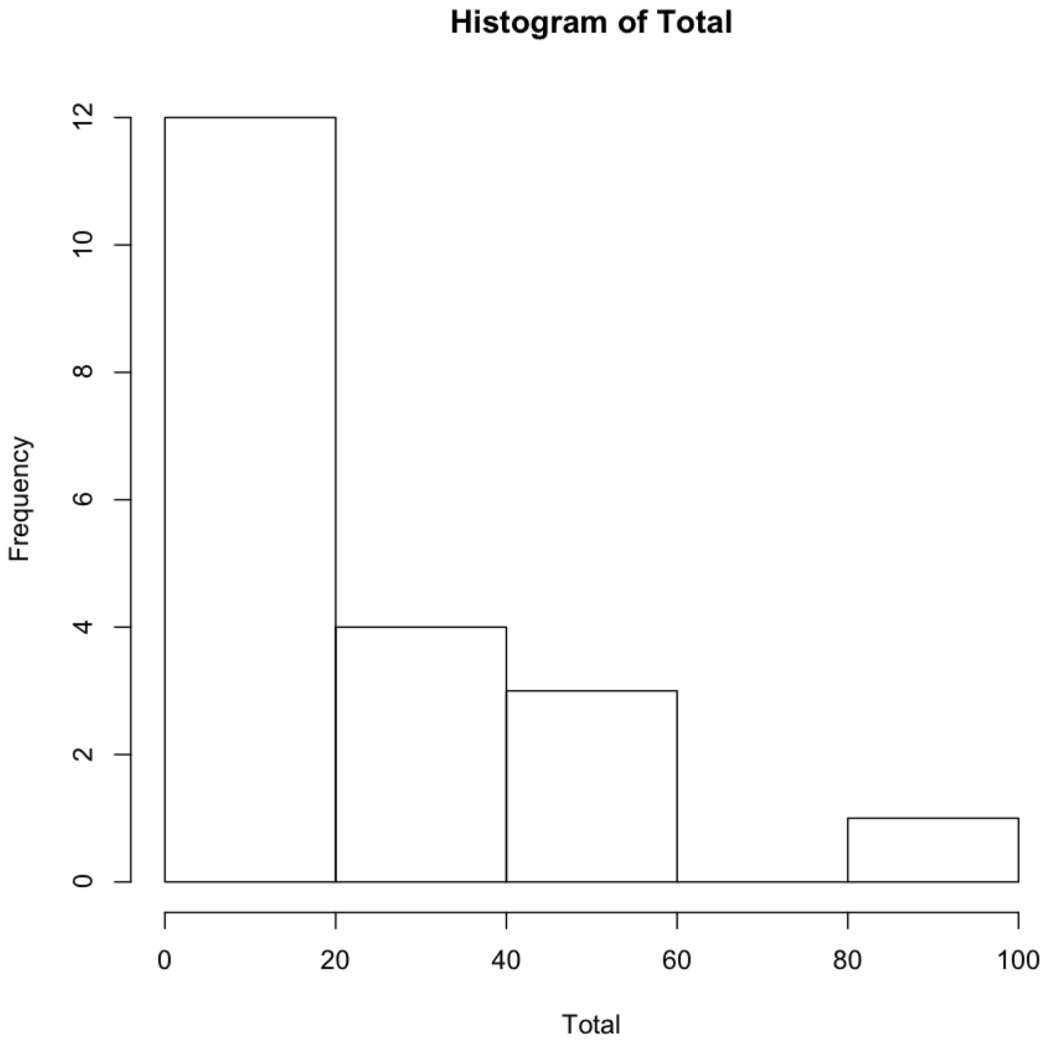

lTotal=log(Total+1):

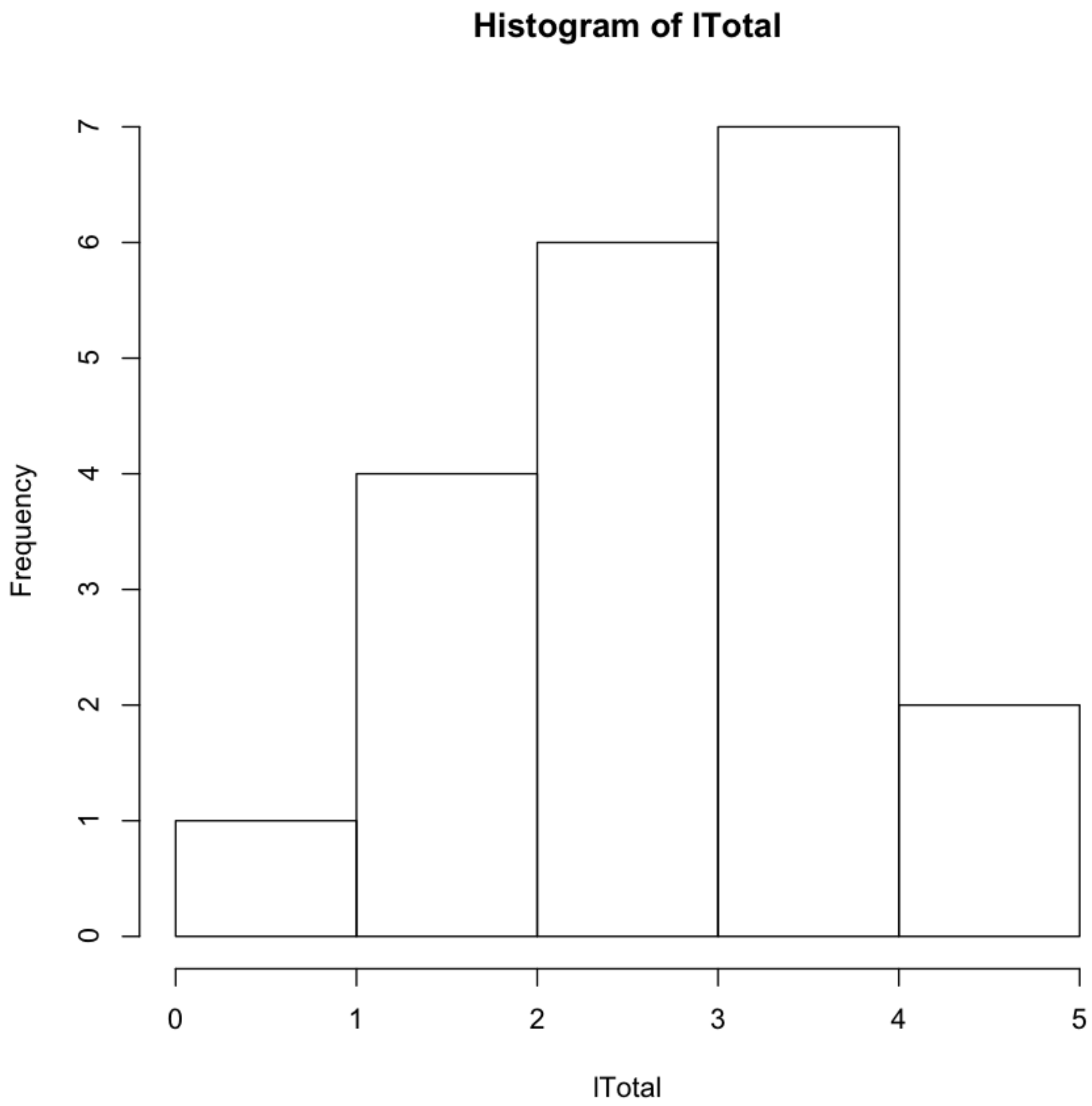

.

fm=lm(lTotal~sites+ WCP + PPP + pH + MO + N + P + K + cl + sand + ashes + CT + N_grass)
> anova(fm)
Analysis of Variance Table

Response: lTotal

	Df	Sum Sq	Mean Sq	F value	Pr(>F)
sites	3	2.7469	0.9156	5.5701	0.065263
WCP	1	2.0907	2.0907	12.7181	0.023454 *
PPP	1	0.5828	0.5828	3.5451	0.132845
pH	1	1.9053	1.9053	11.5902	0.027166 *
MO	1	0.0020	0.0020	0.0120	0.918032
N	1	0.0012	0.0012	0.0072	0.936305

```
P          1    5.1139    5.1139   31.1092    0.005065 **
K          1    0.0971    0.0971    0.5909    0.484959
cl         1    1.3530    1.3530    8.2304    0.045520 *
sand       1    0.1181    0.1181    0.7183    0.444444
ASHES      1    0.9375    0.9375    5.7033    0.075318
CT         1 0.0222  0.0222  0.1348 0.732076
N_grass 1 3.5100  3.5100 21.3525 0.009875 **
Residuals  4 0.6575  0.1644
---
Signif. codes:  0 '***' 0.001 '**' 0.01 '*' 0.05 '.' 0.1 ' ' 1
```

Third Run

```
fm_mixed=lme(lTotal~ WCP + PPP +  pH + MO + N + P + K + cl + sand + ashes + CT +
N_grass,random=~1|sites)

Linear mixed-effects model fit by REML
  Data: NULL
  Log-restricted-likelihood: -23.93365
  Fixed: lTotal ~ WCP + PPP + pH + MO + N + P + K + cl + sand + ashes +      CT +
N_grass
  (Intercept)         WCP         PPP          pH          MO           N
 9.56239925  -0.20954059  0.47615246  0.82853310  0.13106430 -17.22868251
          P            K           cl        sand       ASHES          CT
 0.01808636  -0.88123883  -0.14768821 -0.01247205  -0.56563981  -0.01562934
    N_grass
 2.99484764

Random effects:
 Formula: ~1 | sites
        (Intercept)  Residual
StdDev: 8.196671e-05 0.3971877

Number of Observations: 20

Number of Groups: 4
```
